# Variation and statistical reliability of publicly reported primary care diagnostic activity indicators for cancer: a cross-sectional ecological study of routine data

**DOI:** 10.1136/bmjqs-2017-006607

**Published:** 2017-08-28

**Authors:** Gary Abel, Catherine L Saunders, Silvia C Mendonca, Carolynn Gildea, Sean McPhail, Georgios Lyratzopoulos

**Affiliations:** 1 Primary Care, University of Exeter, Exeter, UK; 2 Cambridge Centre for Health Services Research, University of Cambridge, Cambridge, UK; 3 Knowledge and Intelligence Team (East Midlands), Public Health England, Sheffield, UK; 4 National Cancer Registration and Analysis Service, Public Health England, London, UK; 5 Epidemiology of Cancer Healthcare and Outcomes (ECHO) Group, Department of Behavioural Science and Health, University College London, London, UK

**Keywords:** primary care, health policy, performance measures, quality measurement

## Abstract

**Objectives:**

Recent public reporting initiatives in England highlight general practice variation in indicators of diagnostic activity related to cancer. We aimed to quantify the size and sources of variation and the reliability of practice-level estimates of such indicators, to better inform how this information is interpreted and used for quality improvement purposes.

**Design:**

Ecological cross-sectional study.

**Setting:**

English primary care.

**Participants:**

All general practices in England with at least 1000 patients.

**Main outcome measures:**

Sixteen diagnostic activity indicators from the Cancer Services Public Health Profiles.

**Results:**

Mixed-effects logistic and Poisson regression showed that substantial proportions of the observed variance in practice scores reflected chance, variably so for different indicators (between 7% and 85%). However, after accounting for the role of chance, there remained substantial variation between practices (typically up to twofold variation between the 75th and 25th centiles of practice scores, and up to fourfold variation between the 90th and 10th centiles). The age and sex profile of practice populations explained some of this variation, by different amounts across indicators. Generally, the reliability of diagnostic process indicators relating to broader populations of patients most of whom do not have cancer (eg, rate of endoscopic investigations, or urgent referrals for suspected cancer (also known as ‘two week wait referrals’)) was high (≥0.80) or very high (≥0.90). In contrast, the reliability of diagnostic outcome indicators relating to incident cancer cases (eg, per cent of all cancer cases detected after an emergency presentation) ranged from 0.24 to 0.54, which is well below recommended thresholds (≥0.70).

**Conclusions:**

Use of indicators of diagnostic activity in individual general practices should principally focus on process indicators which have adequate or high reliability and not outcome indicators which are unreliable at practice level.

## Introduction

International variations in cancer survival have been attributed to healthcare system differences in primary care infrastructure.^1^ According to this hypothesis, in systems with a prominent primary care sector, ‘gate-keeping’ by general practitioners results in underutilisation of specialist investigations or referrals, leading to longer diagnostic intervals and poorer clinical outcomes for patients with cancer.^2^ Consequently, increasing attention is being paid to the role of primary care in diagnostic evaluation.^3^

In England, these considerations have led to the development of publicly reported diagnostic activity indicators for general practices (forming part of the Cancer Services Public Health Profile).^4^ Broadly, these include two principal families of indicators: some relating to broader populations of patients most of whom do not have cancer, for example, practice rates of urgent referrals for suspected cancer (hereafter referred to as ‘two week wait’ referrals) or endoscopy tests. Some other indicators relate only to incident cancer cases, for example, the proportion of all cancer diagnoses in a practice that were detected following 2-week wait referrals, otherwise known as ‘detection rate.’ For either class of measures we use the term *activity* (rather than ‘performance’) indicators, given lack of evidence on levels that represent best practice.

Information from the Cancer Services Public Health Profile is used to promote reflective practice, particularly following evidence suggestive of associations between the level of use of 2-week referrals and practice-level cancer survival.^5^ ^6^ However, there are also concerns about the optimal use of practice-level information on these indicators, given uncertainties about their statistical properties and the typically small number of new cancer cases annually in an average practice.^7^ Where small numbers are involved there may be a considerable role of chance, leading to an artefactual inflation of the apparent variation such that, in some cases, most of the observed difference in practice activity may simply reflect chance (box 1).^8^

Box 1 **Inflation of observed variance by chance.**

Where indicators are based on finite numbers, chance will inflate the apparent variation between units of observation. When indicators represent proportions, chance variation will be represented by the binomial distribution, whereas for rate indicators chance variation will be represented by the Poisson distribution. Just as we would not expect practices flipping 10 coins to all record precisely five heads (some will record more and some less by chance), we would not expect every practice with an underlying level of 50% to record exactly that. The effect of chance is larger when the numbers involved are small and it is this phenomenon which is often displayed in funnel plots where the ‘funnel’ is wider at the left of the graph where sample sizes are smaller. Where sample sizes are large, such that the role of chance is small and thus the statistical uncertainty on scores is small, the observed variation will closely approximate the underlying variation.

Against the above background, we characterised the size and sources of variation for diagnostic activity indicators included in the Cancer Services Public Health Profile, and the statistical reliability of potential comparisons of different practices using these measures.

## Methods

In summary, we analysed Cancer Services Public Health Profile data together with information on the age-sex composition of general practice populations to characterise and explain aspects of practice-level variation in diagnostic activity. After describing the observed variation for each diagnostic activity indicator, we used appropriate mixed-effects regression models to estimate the size of the underlying variation between practices. This underlying variation is that which would be seen if the effects of chance were not present. It can be thought of as the variation that would be seen if each practice had a very large sample size (see box 1). These models take into account the role of chance and additionally were used to account for differences in the make-up of practice populations. We further calculated the Spearman-Brown (interunit) reliability of each measure, to assess whether each diagnostic activity indicator included in the ‘Profile’ could reliably distinguish (by which we mean reliably rank or classify) general practices on the basis of their level of diagnostic activity.^1^

### Data

We focused on Cancer Services Public Health Profile for 2013, the most recent year with available data at the time of the analysis. In general, practice indicators (table 1, see online supplementary appendix 1) comprise a ‘numerator’ (eg, the annual number of incident cancer cases, referrals or investigations in a practice), divided by an appropriate denominator (eg, the size of the patient population registered with the practice). Practices with <1000 registered patients are excluded from the public reporting scheme (which resultantly includes information for about 97% of all English general practices), and consequently our analyses.

10.1136/bmjqs-2017-006607.supp1 Supplementary data

**Table 1:** Publicly reported diagnostic process or outcome cancer profile indicators during the study years (see also online supplementary appendices 1 and 3)

Indicator name	Description (proportion or rate)
Process indicators	
Breast screening coverage	Per cent of the eligible practice population (women aged 50–69) screened in the last 36 months
Cervical screening coverage	Per cent of the eligible practice population (women aged 25–64) screened in the target period
Bowel screening coverage	Per cent of the eligible practice population (men and women aged 60–69) screened in the last 36 months
Sigmoidoscopy rate*	Rate per 100 000 registered patients per year
Colonoscopy rate*	Rate per 100 000 registered patients per year
Upper gastrointestinal endoscopy rate*	Rate per 100 000 registered patients per year
TWW referral rate†	Rate per 100 000 registered patients per year
TWW referral rate (colorectal)	Rate per 100 000 registered patients per year
TWW referral rate (lung)	Rate per 100 000 registered patients per year
TWW referral rate (skin)	Rate per 100 000 registered patients per year
TWW referral rate (breast)	Rate per 100 000 registered patients per year
Outcome indicators	
TWW conversion rate	Per cent of TWW referrals resulting in a diagnosis of cancer
TWW detection rate	Per cent of new cancer cases treated which resulted from a TWW referral
Emergency route to diagnosi	Per cent of new cancer cases diagnosed via an emergency hospital admission
Referred route to diagnosi	Per cent of new cancer cases diagnosed following outpatient referral to hospital
Other route to diagnosis	Per cent of new cancer cases diagnosed through another route (eg, via screening)

*For these three (endoscopy) indicators some practices had suppressed data (if count <6), and for those we were able to use numerators imputed with the average among supressed practices.

†The publicly reported data also include an indirectly age-sex standardised rate not used here.

TWW, two-week wait.

We a priori categorised diagnostic activity indicators in those relating to broader populations of patients most of whom will not have cancer (*diagnostic process indicators*)—for example, endoscopy-investigated patients; and those relating to incident cases of cancer (*diagnostic outcome indicators*)—for example, the proportion of patients with cancer diagnosed after an emergency presentation—see also Background.

### Analysis

After exclusions missing data were rare (see online supplementary appendix 3) and a complete case analysis was performed. For each indicator, the analysis comprised four steps.

#### Estimating the observed variation

To summarise the observed variation between general practices (ie, that seen in the publicly reported indicator data), we calculated the variance of the observed practice activity. In order to facilitate a meaningful comparison with modelled variance estimates described below, this variance was calculated on the log odds scale for proportion indicators and the log rate scale for rate indicators.

#### Estimating modelled (underlying) variation

To examine the degree to which observed variation reflects chance, we used a regression model including a random effect for general practice. These models estimate the underlying variance of practice activity (rather than that inflated by chance variation—where the additional chance variation would be described by the binomial or Poisson distributions). Mixed-effects logistic regression was used for ‘proportion’ indicators (eg, screening coverage) and mixed-effects Poisson regression for ‘rate’ indicators (eg, the rate of colonoscopy investigations, table 1). These models contained only a constant term and a random intercept for practice. Importantly, by using these models we are able to handle relatively sparse data (eg, from a single year of data collection) to partition the observed variation into that attributable to chance and that which is underlying variation. These are estimates that are similar to those that would be obtained from observed scores aggregated over many years in a steady state. Practice activity was assumed to be normally distributed on the log odds scale for proportion indicators or log rate scale for rate indicators. Using the outputs of these models, we compared the variance of the modelled (underlying) practice diagnostic activity with that of observed practice scores (as above), to estimate the percentage of the observed variance (in the log odds or log rate scale, as applicable) that is attributable to chance.

#### Estimating modelled variation adjusted for the age-sex profile of practice populations

To examine how much of the observed variation reflects the age and sex make-up of practice populations we repeated the regression models additionally including variables representing proportions of practice populations in different age-sex strata (as we had 18 age groups, 35 such variables were used after excluding one age group-sex stratum which can be determined once all others are known and thus adds no information). From these ‘adjusted-modelled’ analyses, we estimated the variance of practice activity on the log odds or log rate scale as before, adjusted for the age and sex make-up of practice populations. This variance was compared with the variances for the observed and modelled activity to allow respective estimations of the proportion of the observed variance that is due to chance and practice population demography, and the proportion of the modelled variance that is due to practice population demography alone.

#### Distribution of practice activity 

The variances estimated in all above three steps are hard to interpret in respect of the reported indicator values. For this reason, we plotted the distribution of indicator activity in the natural scale. Further, we estimated rate ratio or OR values (as applicable for rate and proportion indicators, respectively) comparing the 75th with the 25th, and the 90th with the 10th centiles of the distributions of practice activity for the observed, modelled and adjusted-modelled distributions. With the observed distribution we used observed centiles of the distribution to calculate these ratios, whereas for the modelled distributions and adjusted-modelled distributions a fully parametric estimation was made using the estimated variance of the random effect and respective percentiles of the normal distribution.

#### Estimating reliability

Finally, we used the same models as for estimating the underlying variation without adjustment for practice age-sex profile to estimate the Spearman-Brown (interunit) reliability of the reported practice activity. Briefly, reliability is a way to assess whether the measured activity for a particular organisation can be meaningfully distinguished from other organisations (ie, whether high and low activity practices for a given indicator can be reliably distinguished from each other).^9^ Reliability is a term often used in USA and UK literature, but the same construct has also been denoted as ‘rankability’ in literature from continental Europe. Further details are included in box 2. A reliability coefficient (taking values from 0—lowest reliability to 1—perfect reliability) is estimated for each practice specific to its denominator and observed activity (see online supplementary appendix 2 for details). The median and IQR of practice reliability is then found along with the number of years of data required for 50% and 90% of practices to reach reliability thresholds of 0.7 and 0.9. These thresholds are used following the examples of others,^9–11^ and while we recognise that any cut-off applied to such statistics is somewhat arbitrary, it has been suggested that the lower threshold (0.7) is desirable for any indicator of quality and the higher threshold (0.9) is preferable for higher stake applications such as pay for performance.^12^ ^10^

All analyses were performed in Stata V.13.1.

Box 2 **Spearman-Brown (or interunit) reliability.**

Spearman-Brown, or interunit, reliability (also known as rankability^9–11 15 24^) is a measure of how reliably units (in this case general practices) can be distinguished (ranked, classified) from each other for a particular indicator. A value of 0 implies that all observed variability is due to chance and so units cannot be distinguished at all from each other on the basis of such an indicator. In contrast a value of 1 implies that all of the observed variability is real and thus all practices can be accurately classified. Reliability is defined as follows:

$$Reliability=\frac{underlying\;between\;unit\;variance}{underlying\;between\;unit\;variance+\frac{within\;unit\;variance}{n}}$$

where n is the number of observations for a given practice. Often reliability is thought of as the proportion of observed variance that is attributable to the underlying variance (alternatively, the proportion of overall variance *not* due to chance). However, in ascribing a single reliability value to all units we might suggest all have the same level of distinguishability. Reliability of binary and rate indicators depends on three factors:

- unit sample size, with a higher sample size leading to more precise unit score estimates and thus increasing reliability;
- unit score, with percentage scores closer to 50% leading to smaller within-unit variances on the log odds scale for the same sample size, thus leading to more precise unit score estimates and thus increasing reliability;
- between-unit variance, with larger between-unit variances making it easier to distinguish units with the same absolute precision of estimated score, thus increasing reliability.

Given variation in score and sample size between units we follow the example of Adams *et al* and consider reliability to be unit specific rather than a single value applying to the whole population.^11^ For this reason, the median reliability values shown in table 2 do not relate directly to the proportion of variance of observed activity attributable to chance in table 3. The latter are heavily influenced by smaller practices with much larger variance due to chance.

## Results

Between 7687 and 7954 practices were included in the analysis (table 2, see online supplementary appendix 3). The mean list size of these practices was 7034 (SD 4293) ranging from 1012 to 46 126. The median annual number of new cancer cases per practice was 28 (IQR 15–48). Diagnostic process indicators (relating to tested or referred patients) and diagnostic outcome indicators (relating to patients with incident cancer) had notably different number of cases or events per practice. For example, the median annual number of persons screened for colorectal cancer (relating to the ‘bowel screening coverage’ diagnostic process indicator) was 349 per practice; while the median annual number of cancer cases detected after 2-week wait referrals (relating to ‘detection rate’ diagnostic outcome indicator) was 12 per practice (table 2).

**Table 2:** Number of practices by diagnostic process or outcome indicator, median (IQR) activity, indicator reliability for practice activity at the median (50th), and the 25th and 75th centiles of the distribution and years required for reliability of 0.7 and 0.9

Indicator	Number of practices included	Median/IQR cases/events per practice	Median/IQR practice indicators		Median (IQR) Spearman-Brown reliability	Years required for reliability 0.7		Years required for reliability 0.9	
						50% of practices	90% of practices	50% of practices	90% of practices
Diagnostic process indicators									
Breast screening coverage	7951	502 (258–862)	71.3 (63.9–76.3)	%	0.96 (0.93–0.97)	0.1	0.3	0.4	1.0
Cervical screening coverage	7910	1122 (644–1744)	74.9 (70.3–78.5)	%	0.97 (0.95–0.98)	0.1	0.2	0.3	0.8
Bowel screening coverage	7924	349 (166–632)	57.0 (48.6–62.7)	%	0.96 (0.93–0.97)	0.1	0.3	0.4	1.2
Sigmoidoscopy rate	7954	24.5 (13–44)	4.1 (2.8–5.6)	Per 1000 person-years	0.86 (0.76–0.91)	0.4	1.2	1.5	4.8
Colonoscopy rate	7954	39 (20–65)	6.4 (4.7–8.4)	Per 1000 person-years	0.87 (0.79–0.92)	0.3	1.1	1.3	4.1
Upper gastrointestinal endoscopy rate	7954	66 (35–110)	10.8 (8.3–13.8)	Per 1000 person-years	0.91 (0.84–0.94)	0.2	0.7	0.9	2.7
TWW referral rate	7954	124 (60–219)	20.0 (14.2–26.4)	Per 1000 person-years	0.97 (0.93–0.98)	0.1	0.3	0.3	1.2
TWW referral rate (colorectal)	7954	21 (9–38)	3.4 (2.0–4.8)	Per 1000 person-years	0.88 (0.77–0.93)	0.3	1.4	1.3	5.6
TWW referral rate (lung)	7954	5 (2–9)	0.8 (0.4–1.2)	Per 1000 person-years	0.60 (0.45–0.72)	1.6	4.8	6.1	18.3
TWW referral rate (skin)	7954	20 (8–37)	3.2 (1.8–4.8)	Per 1000 person-years	0.90 (0.80–0.94)	0.3	1.2	1.0	4.7
TWW referral rate (breast)	7954	22 (11–39)	3.7 (2.5–4.9)	Per 1000 person-years	0.81 (0.68–0.88)	0.6	2.0	2.2	7.7
Diagnostic outcome indicators									
TWW conversion rate	7954	12 (6–22)	9.9 (7.2–13.0)	%	0.54 (0.37–0.67)	2.0	7.6	7.6	29.2
TWW detection rate36	7941	12 (6–22)	47.1 (38.2–55.6)	%	0.32 (0.19–0.45)	5.0	16.8	19.3	64.6
Emergency route to diagnosis	7687	6 (3–11)	23.4 (17.2–30.8)	%	0.24 (0.14–0.33)	7.6	24.6	29.1	94.7
Referred route to diagnosis	7687	13 (6–22)	49.2 (40.0–56.3)	%	0.22 (0.13–0.32)	8.2	28.1	31.5	108.5
Other route to diagnosis	7687	7 (3–12)	26.1 (19.1–33.9)	%	0.33 (0.21–0.45)	4.8	15.6	18.6	60.2
Other indicators									
Cancer mortality	7954	13 (7–23)	2.3 (1.5–2.9)	Per 1000 person-years	0.70 (0.55–0.80)	1.0	3.3	3.9	12.5
Emergency cancer hospitalisations	7935	28 (15–48)	4.7 (3.4–6.0)	Per 1000 person-years	0.81 (0.70–0.88)	0.5	1.7	2.1	6.6
Incident cases	7946	28 (15–48)	4.8 (3.4–6.0)	Per 1000 person-years	0.80 (0.68–0.87)	0.6	1.9	2.2	7.2
Prevalent cases	7960	112 (55–192)	1.9 (1.4–2.4)	%	0.96 (0.92–0.97)	0.1	0.4	0.4	1.4

TWW, two-week wait.

### Between-practice variation and the role of chance and that of practice population demography

Overall the size of modelled (underlying) practice variation was substantial, with typically up to twofold variation between practices at the 75th and the 25th centiles of the distribution, and up to fourfold variation between practices at the 90th and 10th centiles (table 3, *columns 7 and 10*).

**Table 3:** Size of variation, and proportions of observed variance explained by chance and/or age-sex differences between practice populations. Note that the per cent of variance explained is on the log/log odds, respectively, as opposed to the natural scale

Column 1	Column 2	Column 3	Column 4	Column 5	Column 6	Column 7	Column 8	Column 9	Column 10	Column 11
Diagnostic process or outcome indicators	Number of practices included	Per cent of variance due to chance alone	Per cent of variance due to practice age-sex profile (beyond role of chance)	Per cent of variance due to both chance and practice age-sex profile	Observed (actually reported) 75th/25th RR/OR	Adjusted for chance 75th/25th RR/OR	Adjusted for chance and practice age-sex profile 75th/25th RR/OR	Observed (actually reported) 90th/10th RR/OR	Adjusted for chance 90th/10th RR/OR	Adjusted for chance and practice age-sex profile 90th/10th RR/OR
Process indicators										
Breast screening coverage	7951	9.5	53.8	58.2	1.82	1.75	1.46	3.03	2.90	2.06
Cervical screening coverage	7910	6.9	46.0	49.8	1.54	1.57	1.39	2.36	2.37	1.88
Bowel screening coverage	7924	13.9	75.3	78.8	1.78	1.70	1.30	2.92	2.74	1.65
Sigmoidoscopy rate	7954	31.1	24.9	48.3	1.99	1.93	1.77	4.22	3.52	2.97
Colonoscopy rate	7954	37.8	38.6	61.8	1.81	1.76	1.56	3.26	2.96	2.34
Upper gastrointestinal endoscopy rate	7954	26.5	33.9	51.4	1.67	1.70	1.54	2.85	2.74	2.27
TWW referral rate	7954	19.6	39.9	51.7	1.86	1.92	1.66	3.56	3.46	2.62
TWW referral rate (colorectal)	7954	34.1	41.4	61.4	2.36	2.17	1.81	6.44	4.40	3.11
TWW referral rate (lung)	7954	40.4	25.0	55.3	2.92	2.03	1.85	12.56	3.87	3.23
TWW referral rate (skin)	7954	27.1	31.9	50.3	2.67	2.45	2.10	8.59	5.55	4.11
TWW referral rate (breast)	7954	45.0	20.1	56.1	1.98	1.78	1.68	4.07	3.02	2.69
Outcome indicators										
TWW conversion rate	7954	64.8	30.6	75.6	1.92	1.55	1.44	3.93	2.31	2.01
TWW detection rate	7941	78.4	4.9	79.5	1.98	1.43	1.42	4.25	1.99	1.95
Emergency route to diagnosis	7687	82.8	30.1	88.0	2.15	1.39	1.32	5.09	1.89	1.70
Referred route to diagnosis	7687	85.0	7.4	86.1	1.92	1.32	1.30	3.64	1.69	1.66
Other route to diagnosis	7687	74.8	12.5	77.9	2.13	1.50	1.47	5.11	2.18	2.08

RR, risk ratio; OR, odds ratio;

TWW, two-week wait.

The proportion of overall variance in the observed activity that is due to chance ranged from 7% to 45% for diagnostic process indicators and from 65% to 85% for diagnostic outcome indicators (table 3, *column 3*). After accounting for variation due to chance, variation attributable to the age-sex profile of practice population ranged from 20% to 75% for diagnostic process indicators and from 5% to 31% for diagnostic outcome indicators (table 3, *column 4*). The combined proportion of variation due to both chance and practice age-sex profile ranged from 48% to 79% for diagnostic process and from 76% to 88% for diagnostic outcome indicators (table 3, *column 5*).

#### Visualising variation

**Figure 1:**
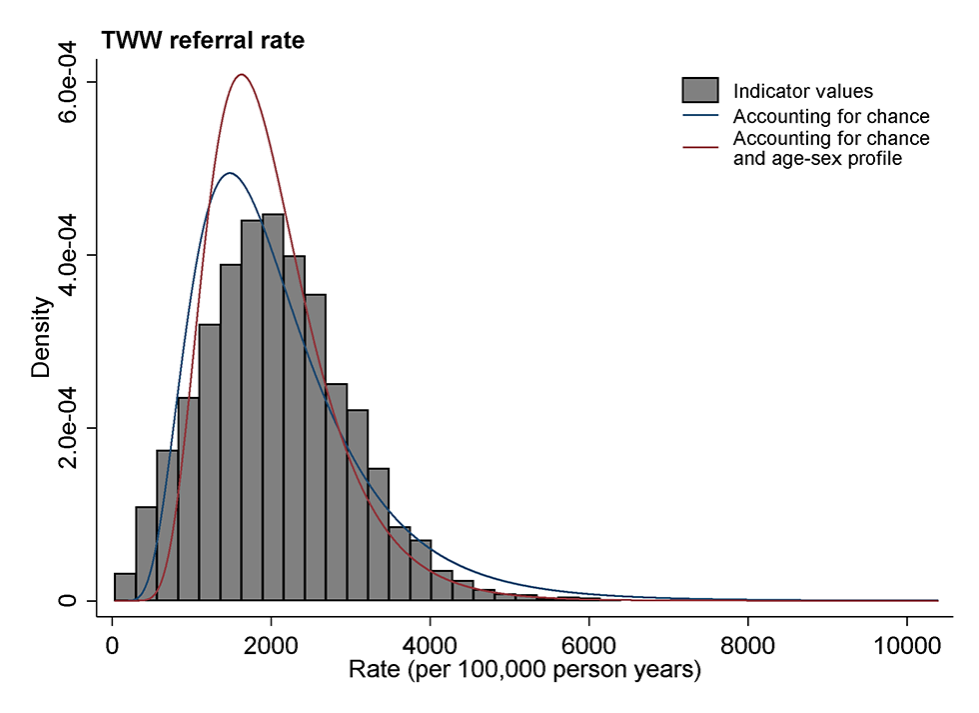
Illustration of the distribution of a diagnostic process indicator (2-week wait referral) for general practices. Observed values are denoted in grey histogram bars. The distribution of underlying practice activity, accounting for chance, is denoted with a blue curve line. The distribution of underlying practice activity adjusting for age-sex differences in practice populations is denoted by a red curve line. TWW, two-week wait.

**Figure 2:**
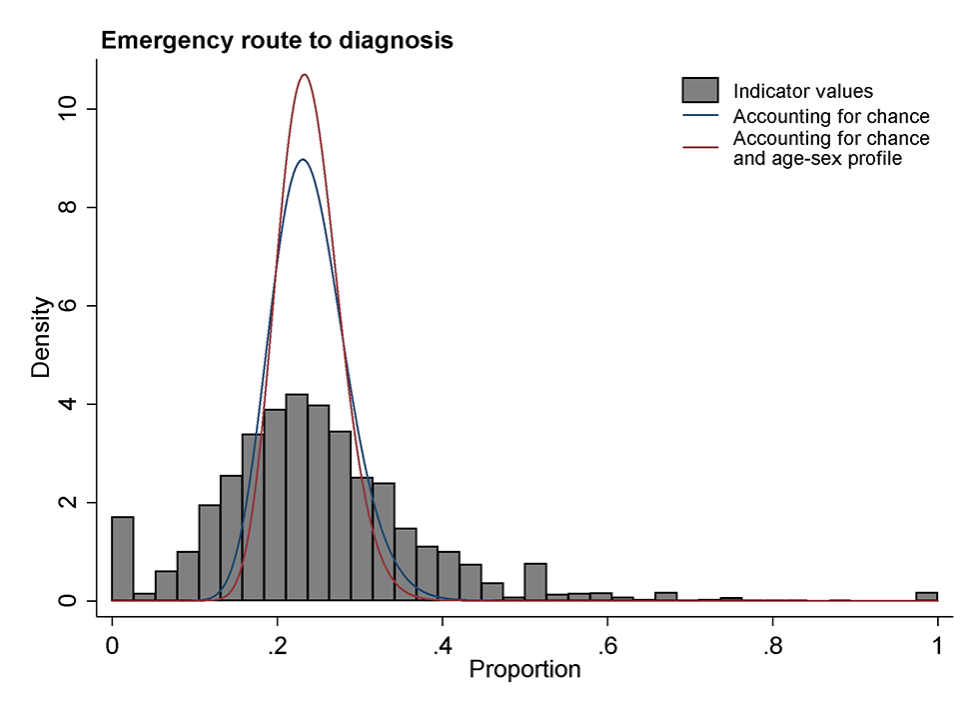
Illustration of the distribution of a diagnostic outcome indicator (per cent of cancer cases diagnosed following an emergency presentation) for general practices. Observed values are denoted in grey histogram bars. The distribution of underlying practice activity, accounting for chance, is denoted with a blue curve line. The distribution of underlying practice activity adjusting for age-sex differences in practice populations is denoted by a red curve line.

In figures 1 and 2, we illustrate differences between the observed, modelled and adjusted-modelled variations for a diagnostic process indicator (the rate of 2-week wait referrals) and an outcome indicator (the proportion of all new cancer diagnoses in a practice diagnosed through an emergency presentation).

For 2-week wait referrals (figure 1), the spread of observed distribution (illustrated by the histogram) is similar to that of the modelled distribution accounting for chance (denoted by a blue line), although the latter is slightly narrower. This reflects the difference between observed and modelled variances for this indicator, which is 20%, one of the smallest among all indicators (table 3, *column 3*).

Conversely, for the proportion of patients with cancer diagnosed as emergencies (figure 2), the spread of the observed variation is far wider than that of the modelled variation, indicating that the observed differences are grossly inflated by chance. This also reflects that a very large proportion (ie, 83%) of the observed variation for this indicator is due to chance (table 3, *column 3*). It is worth noting that the peaks in the observed distribution at 0 and 0.5 are dominated by practices with very few emergency presentations (<5).

In both figure 1 and figure 2 the distributions shown by red lines (ie, accounting for both chance and the age-sex profile of practice population) are somewhat narrower than the distributions shown by blue lines (which simply account for chance). These differences reflect the 40% and 30% of variance beyond chance explained by the age-sex profile of practice in these two indicators, respectively (table 3, *column 4*).

### Indicator reliability

The reliability of the publicly reported indicators included in the ‘Cancer Services Public Health Profile’ was bimodal: diagnostic outcome indicators relating to incident cancer cases (eg, rate of 2-week wait referrals, or the per cent of cancer cases presenting as emergencies) have low reliability, well below thresholds required for high-stake applications and performance management (≥0.90) and, indeed, lower than thresholds of merely adequate reliability (≥0.70). These include conversion (proportion of all 2-week wait referrals resulting in a cancer diagnosis) and detection rates (proportion of all new cancers treated which resulted from a 2-week wait referral), with median reliabilities of 0.54 and 0.32, respectively (table 2). They also include the proportions of patients diagnosed via various routes (emergency, elective and ‘other’) which have median reliabilities of ≤0.33. Reliable estimates could be achieved by using multiple years of data for these indicators, but often too many to make this practical (eg, more than 24 years to achieve a reliability of at least 0.7 for 90% of practices for the proportion of patients diagnosed via an emergency route, table 2).

This contrasts with diagnostic process indicators relating to broader populations (eg, patients investigated by endoscopy). Reliability is particularly high for all three screening coverage indicators (r≥0.96); all three endoscopy activity indicators (r≥0.86) and also for most 2-week wait referral indicators (any type r=0.97, colorectal, breast and skin cancer, r>0.80 for all three) (table 2). The single diagnostic process indicator with inadequate reliability is the 2-week wait referral indicator for suspected lung cancer (r=0.60) which, unusually among process indicators, on average relates to only few (5) cases per practice.

## Discussion

### Summary of main findings

There is substantial variation between English general practices in respect of referral or investigation activity of relevance to cancer diagnosis, even after the roles of chance and the age-sex profile of practices are taken into account. However, practices can only be reliably classified for their propensity for high or low activity in respect of diagnostic process indicators (eg, 2-week referrals, endoscopy use or screening programme coverage). In contrast, diagnostic outcome indicators relating to incident cancer cases (eg, conversion or detection rates, or the proportion of cancers diagnosed as emergencies) are not reliable and practices should not be ranked in respect of such indicators. Reliability is driven by three factors: sample size, score and between-practice variance. In general, the poor reliability of diagnostic outcome indicators reflects the small sample size (see box 2). An exception to this general pattern is cancer mortality in a general practice population, which is an indicator with small numbers but modest reliability. It should be noted that mortality due to cancer in a general practice is a measure of disease burden and the relatively high reliability reflects larger variability between practices due to substantial sociodemographic differences in practice populations.

### Comparison with the literature

We are not aware of prior studies specifically examining variation and reliability of a wide range of practice indicators included in the General Practice Profiles for Cancer. Observed variation in a small number of these indicators has been described previously, although previous studies have not formally examined the degree of variation due to mere chance.^13^ ^14^ A recent study from Scotland has attributed much of the variation in three indicators of referral activity (2-week wait referrals, and resultant conversion and detection rates) to ‘random case-mix.’^7^ In that paper, all chance variation is ascribed to variability in the case mix of presenting patients.^7^ If case mix was dominating the observed variation between practices then this could be overcome by adjusting or standardising for case mix. However, as shown by our study, chance variation would exist even in the absence of variable case mix. By examining a wide range of diagnostic evaluation measures included in our study (eg, regarding practice-level endoscopy use and screening programme coverage) and by quantifying the reliability of the indicators, we have shown that not all diagnostic activity indicators in current use are dominated by chance, and reporting could concentrate on those which are reliable. More broadly, our study adds to the prior literature on reliability and variability for organisational comparisons. Much of the prior literature relates to the context of hospital indicators where sample sizes are often larger.^15^ ^16^ ^17^ ^18^ ^19^ Previous studies described variation and/or the reliability of other indicators of general practice activity or quality chiefly considering patient experience, or prescription or cost patterns, but not diagnostic use indicators.^9^ ^10^ ^20^ ^21^ ^11^ ^22^ As has been remarked previously, the reliability of process measures is generally substantially higher than that of outcome measures, as the former encompass broader populations of patients with and without the disease or outcome of interest, whereas the latter relate to smaller populations of actual cases.^23^ Of course this is a generalisation, and it may be the case that outcome indicators are reliable, for example, a Dutch study found unintended reoperation after colorectal surgery to have an acceptable level of reliability.^24^

### Strengths and limitations

We have used appropriate modelling techniques to separately account for the role of chance and the influence of differences in the age-sex make-up of practice populations, within the limits of the ecological data used. Our methods adjust for practice demography but do not account for the individual characteristics of patients who were referred or investigated. Individual level adjustment would have been preferable to fully adjust for confounding that may occur at patient level, but individual level case-mix data are not routinely collected as part of some of the respective data sets.^25^ By taking practice demography into account we may have overadjusted as, for example, patients of the same age may experience different diagnostic or referral activity in practices with averagely older populations.

Our reliability estimates appropriately relate to practice activity as it is publicly reported, which, with one exception, do not take into account individual case mix or the age-sex profile of practice populations. Where indicators are used for purposes of service planning or resource allocation, information is needed on observed as opposed to adjusted indicators. Where practice demography is the dominant source of variation, the reliability without adjustment for demography may simply reflect differences in the make-up of practice populations rather than real differences in clinical practice. In other words, such indicators may reliably distinguish practices with fewer older patients from those with more.

We have a priori not taken into account the deprivation scores of practice populations—because deprivation may be a mediator rather than a confounder of practice performance. We were not able to encompass practitioner-level variation in use of diagnostic tests or referrals—such a study is impossible to perform as data on diagnostic activity generated by individual doctors are not currently collected routinely (only data aggregated at practice level exist). However, other evidence indicates that the size of within-practice variation (ie, between the GPs of the same practice) is non-ignorable.^26^

### Implications

Our study has several implications for policy and research. The fact that there is non-ignorable variation between practices (beyond that driven by chance and the demographic characteristics of the patient populations they serve) should motivate further inquiries about reasons likely to be responsible for this variation. This can take two approaches. First, studies that will examine potential correlations of diagnostic activity with other measures of practice quality and/or practice characteristics. Second, qualitative (including ethnographic and significant event audit) studies that will examine contextual and compositional (doctor or patient-related) factors determining diagnostic activity.

In their present form, ‘Practice profiles’ characterise practice scores reliably for some indicators but not others. These findings can be considered in respect of two possible applications. The first is one where all practices were ranked against each other for their position in the national distribution. As we have shown, for unreliable diagnostic outcome indicators such rankings will be dominated by chance and are unlikely to reflect underlying diagnostic activity. In contrast, reliable process indicators are well suited to such use. Second, individual practices can be compared with a national mean. Normally, uncertainty in practice estimates is taken into account when such comparisons are being made, and only practices where the difference from the national mean is statistically significant are flagged. While this obviates the problem of unreliably comparing practices against each other, there is still a high risk of type II error for unreliable indicators, meaning that many practices whose underlying activity is substantially different to the mean will not be identified as such. Several options exist to address the latter problem. For example, indicators with inadequate median reliability (ie, <0.70) can either be excluded from the reporting scheme, or be appropriately ‘flagged’ so that their interpretation and use (by healthcare professionals or managers, or members of the public) can be appropriately informed. The latter convention has been adapted by the public reporting of the ‘Practice Profiles’ recently (2015).

Evidence nonetheless indicates that greater use by general practices of 2-week wait referrals, and greater use of upper gastrointestinal endoscopies are associated with better clinical outcomes.^5^ Examining potential associations between clinical outcomes and other types of diagnostic activity (other than 2-week wait referrals and upper gastrointestinal endoscopy) would be useful. Future research should also address the optimal levels of diagnostic or referral activity from the perspective of optimising population health outcomes and resource utilisation.

In conclusion, we identify substantial variation in practice level diagnostic processes and outcomes relating to cancer (beyond those that could be expected by chance or the age-sex profiles of practice populations), suggesting meaningful improvements are possible. Information on diagnostic outcome indicators has low reliability, therefore patients, clinicians and managers should avoid judging practices against these indicators. In contrast, diagnostic process indicators are statistically reliable and could be used to classify practice activity. The findings should motivate further research addressing the causes and consequences of variation in indicators of diagnostic evaluation in primary care. Although we examined practice indicators of diagnostic activity relevant to cancer, the findings could be relevant to other publicly reported primary care indicators, particularly if they relate to small number of cases per practice. Addressing the challenge of reliable measurement and public reporting of organisational or provider activity requires both further empirical work and a culture change among patients, practitioners and policymakers.
