# Supplementary material for: Variation and statistical reliability of publicly reported primary care diagnostic activity indicators for cancer: a cross-sectional ecological study of routine data
Source: BMJ Qual Saf. 2017 Aug 28;27(1):21–30. doi: 10.1136/bmjqs-2017-006607 (PMC5750427; doi:10.1136/bmjqs-2017-006607)
Supplement: Supplementary data [file bmjqs-2017-006607supp001.pdf]

Appendix 1a – Data sources and specification

Characterising the population of each practice. For all general practice populations included in the analysis, denominator information [practice list size, and practice population age (in five year age groups) and sex profiles] were obtained for each practice from the Attribution Data Set from the English Exeter GP Registration System which is extracted once a year. Data for 2008-10 practice populations size and characteristics are available from publicly available Practice Based Commissioning data sets[4] and for 2011-13 data was provided directly to the study authors by the East of England Public Health England Knowledge and Intelligence Team.

Indicator outlier values

Initial analysis identified some outlier values for the screening uptake indicators. These were identified visually from the distributions. In all three cases there was a very long tail towards highly unlikely low uptake values, inconsistent with the variability seen for the remaining practices. In some cases there was even an indication of a secondary peak at these low values. These low values existed in the presence of large denominators indicating they were unlikely to be due to chance. A cut-off was chosen following visual inspection (Appendix 3).

Appendix 1b. Data sources and related periods

Indicator	Data Source	Date
**Process**		
Breast screening coverage	Exeter	April 2013†
Cervical screening coverage	Exeter	April 2013†
Bowel screening coverage	Exeter	April 2013†
Sigmoidoscopy rate	HES	April 2012 to March 2013*
Colonoscopy rate	HES	April 2012 to March 2013*
Upper GI endoscopy rate	HES	April 2012 to March 2013*
TWW referral rate	CWT	April 2012 to March 2013*
TWW referral rate (Colorectal)	CWT	April 2012 to March 2013*
TWW referral rate (Lung)	CWT	April 2012 to March 2013*
TWW referral rate (Skin)	CWT	April 2012 to March 2013*
TWW referral rate (Breast)	CWT	April 2012 to March 2013*
**Outcome**		
TWW conversion rate	CWT	April 2012 to March 2013
TWW detection rate	CWT	April 2012 to March 2013
Emergency route to diagnosis	RTD	January to December 2008
Referred route to diagnosis	RTD	January to December 2008
Other route to diagnosis	RTD	January to December 2008

†Denominators used for these indicators are the number of eligible patients in the practice April 2013
* Denominators used for these indicators are the practice list size taken from QOF 2012/13 QOF data which is defined as the list size at January 2013

TWW; two week wait, Exeter; the Exeter database, ONS; Office of National Statistics, QOF; Quality and Outcomes Framework, HES; Hospital Episode Statistics, CWT; Cancer Waiting Times database, RTD; Routes to Diagnosis database

Appendix 2 – Estimating reliability

Unit level, or Spearman Brown, reliability is given by

$$\text{Reliability} = \frac{\text{underlying between unit variance}}{\text{underlying between unit variance} + \frac{\text{within unit variance}}{n}}$$

Where n is the number of observations per unit and in the case of binary or rate indicators the within unit variance is assumed to follow the binomial or Poisson distribution respectively. In the context of this study a unit is a practice, but we use the terminology unit here to be more general.

Following on from the definition above, reliability is often estimated by first estimating the between unit variance and the within unit variance. However, in the context of the binomial or Poisson distribution estimating the within unit variance is not straightforward. Although various methods have been proposed to estimate the within unit variance we employ a method which does not directly use variance estimates. Instead we utilise the relationship between “Empirical Bayes” estimates of Unit score and Maximum Likelihood estimates of the unit score. “Empirical Bayes” estimates of unit scores (also known as Best Linear Unbiased Predictions or BLUPs) are related to the observed scores (Maximum Likelihood estimates) through reliability. Specifically the observed scores (on the appropriate scale) are shrunk towards the mean of unit scores by an amount equal to the inter-unit reliability. Thus by knowing both the “Empirical Bayes” estimates of Unit scores and Maximum Likelihood estimates of the unit score we can obtain an estimate of reliability for each Unit.

The first step in estimating unit reliabilities is to fit a mixed-effect generalised linear model which contains only a constant term and a random intercept for unit, i.e.

$$\eta_i = \beta_0 + s_i$$

In the case of proportion indicators $\eta_i = \text{logit}(\pi_i)$, where π_i is the underlying proportion in unit i and the data within unit are assumed to be binomially distributed. In the case of rate indicators $\eta_i = \log(\lambda_i)$, where λ_i is the underlying rate in unit i and the data within unit are assumed to follow the Poisson distribution. In each case s_i represents a unit effect and is assumed to be normally distributed with a mean of zero, and β_0 is a constant term, both of which are on the log-odds scale for proportion indicators and the log-rate scale for rate indicators. Following fitting of the model, “Empirical Bayes” estimates of unit effects, $\hat{s}_i^{EB}$, are obtained which represent the best estimate of the deviation of unit i from the mean of all units β_0.

The estimated inter-unit reliability for proportion indicators is given by

$$\text{Reliability}_i = \frac{\hat{s}_i^{EB}}{\text{logit}(\hat{\pi}_i) - \beta_0}$$

And for rate indicators is given by

$$\text{Reliability}_i = \frac{\hat{s}_i^{EB}}{\log(\hat{\lambda}_i) - \beta_0}$$

Where $\hat{\pi}_i$ and $\hat{\lambda}_i$ are the observed proportion or rate in unit i respectively.

Initial work showed that for binary indicators, reliability estimated in this way was indistinguishable from that estimated using the method applied by Lawson et al.[1]

1. Lawson EH, Ko CY, Adams JL, Chow WB, Hall BL. Reliability of evaluating hospital quality by colorectal surgical site infection type. Ann Surg. 2013;258(6):994-1000.

Appendix 3. Details of practice exclusions and final number of included practices for each indicator.

**Indicators**	**Number of 2013 practice profiles practices**	Number of profiles with missing numerators	Number of practices with missing denominator	Number of practices with zero denominators	Number of practices with missing practice population age/sex information	Outlier indicator values	**N (%) practices not included**	**Total number of practices included**
Process indicators								
Breast screening coverage	7962	2	2	0	0	7	11 (0.1)	7951
Cervical screening coverage	7962	20	10	0	1	21	52 (0.7)	7910
Bowel screening coverage	7962	37	0	0	0	1	38 (0.5)	7924
Sigmoidoscopy rate	7962	0	0	n/a	8	0	8 (0.1)	7954
Colonoscopy rate	7962	0	0	n/a	8	0	8 (0.1)	7954
Upper GI endoscopy rate	7962	0	0	n/a	8	0	8 (0.1)	7954
TWW referral rate	7962	1	0	n/a	7	0	8 (0.1)	7954
TWW referral rate (Colorectal)	7962	1	0	n/a	7	0	8 (0.1)	7954
TWW referral rate (Lung)	7962	1	0	n/a	7	0	8 (0.1)	7954
TWW referral rate (Skin)	7962	1	0	n/a	7	0	8 (0.1)	7954
TWW referral rate (Breast)	7962	1	0	n/a	7	0	8 (0.1)	7954
Outcome indicators								
TWW conversion rate	7962	1	0	0	7	0	8 (0.1)	7954
TWW detection rate	7962	1	0	14	6	0	21 (0.3)	7941
Emergency route to diagnosis	7962	55	0	87	133	0	275 (3.5)	7687
Referred route to diagnosis	7962	55	0	87	133	0	275 (3.5)	7687
Other route to diagnosis	7962	55	0	87	133	0	275 (3.5)	7687
Other indicators								
Cancer mortality	7962	0	0	n/a	8	0	8 (0.1)	7954
Emergency cancer hospitalisations	7962	26	0	n/a	1	0	27 (0.3)	7935
Incident cases	7962	0	0	n/a	16	0	16 (0.2)	7946
Prevalent cases	7962	0	0	0	2	0	2 (0.0)	7960
